# Determining Access for a City‐Wide Extracorporeal Cardiopulmonary Resuscitation (ECPR) Initiative Using Geospatial Analysis

**DOI:** 10.1111/acem.70288

**Published:** 2026-04-13

**Authors:** Christiana K. Prucnal, Melissa A. Meeker, Rebecca E. Cash, Erica L. Nelson, P. Gregg Greenough, Stephen D. Hallisey, Annette M. Ilg, Christopher Kabrhel, Raghu R. Seethala, Paul S. Jansson

**Affiliations:** ^1^ Department of Emergency Medicine Harvard Medical School Boston Massachusetts USA; ^2^ Department of Emergency Medicine Brigham and Women's Hospital Boston Massachusetts USA; ^3^ Department of Emergency Medicine Massachusetts General Hospital Boston Massachusetts USA; ^4^ Division of Emergency Critical Care Medicine, Department of Emergency Medicine Brigham and Women's Hospital Boston Massachusetts USA; ^5^ Division of Critical Care Medicine, Department of Emergency Medicine University of Michigan Ann Arbor Michigan USA; ^6^ Division of Pulmonary and Critical Care Medicine, Department of Internal Medicine University of Michigan Ann Arbor Michigan USA

**Keywords:** emergency medical services (EMS), extracorporeal cardiopulmonary resuscitation (ECPR), extracorporeal membrane oxygenation (ECMO), geographic information systems (GIS), out of hospital cardiac arrest (OHCA), spatial analysis, traffic network patterns

## Abstract

**Background:**

In select situations, patients experiencing out‐of‐hospital cardiac arrest (OHCA) may be candidates for extracorporeal cardiopulmonary resuscitation (ECPR). Eligibility criteria for ECPR typically include a maximum time (usually 30 min) from arrest to arrival at an ECPR‐capable center, which may exclude populations based on geographic factors.

**Methods:**

Using geospatial modeling, we calculated drive times to ECPR‐capable hospitals in Boston utilizing census block group centroid coordinates as proxy sites for OHCA locations. We used a fixed dispatch‐to‐scene arrival time of 7.4 min, extrapolated from Boston EMS median transport time data. We set conditions at the 50th (24 min), 25th (18 min), and 10th (13 min) percentiles for EMS on‐scene time and, for each condition, determined access to ECPR with an arrest to arrival criterion of less than 30 min. We analyzed the effect of high‐ versus low‐traffic conditions and then derived the arrest to arrival time necessary to achieve access for 90% of the city.

**Results:**

The entire City of Boston was excluded from ECPR with median times and current eligibility criteria. Decreasing time‐on‐scene to the 25th percentile led to increased access: 16% of block groups with low traffic and 6% of block groups with high traffic. At the 10th percentile for time‐on‐scene, 55% of block groups had access with low traffic and 28% had access with high traffic. To achieve access for 90% of the city under high‐traffic conditions at the 50th percentile for time‐on‐scene, the criterion for arrest to arrival would need to be extended to 55.8 min.

**Conclusions:**

The current arrest to arrival criterion for ECPR excludes the entire City of Boston using median transportation and on‐scene times. Increasing access to ECPR should include efforts to decrease prehospital duration, such as minimizing time‐on‐scene for potential OHCA cases. Future study should examine potential levers to improve access, such as novel prehospital ECPR delivery models, air‐based transport, and liberalized arrest to arrival criteria.

## 1 Background

Survival and functional recovery after out of hospital cardiac arrest (OHCA) remain low [1]. Although significant strides have been made, including increased bystander cardiopulmonary resuscitation (CPR), automated external defibrillator (AED) use, and double sequential external defibrillation, apart from high quality CPR with minimal interruptions, few interventions have been shown to improve outcomes in this population [2, 3, 4]. Veno‐arterial extracorporeal membrane oxygenation (VA‐ECMO, hereafter ECMO) is a highly invasive technology that serves as a heart and lung bypass machine. While available since the 1960s, ECMO has only recently been studied as an adjunct life support measure in patients with OHCA, particularly those in refractory ventricular fibrillation (VF) or ventricular tachycardia (VT) [5]. Under certain circumstances, ECMO may be considered for highly selected patients in cardiac arrest to maintain perfusion, oxygenation, and ventilation as a bridge to definitive treatment of potentially reversible causes, and this intervention is termed extracorporeal cardiopulmonary resuscitation (ECPR) [6]. In preliminary studies and early controlled trials, ECPR has been associated with improved survival and outcomes in cardiac arrest, although additional data are still needed [7, 8, 9, 10, 11, 12, 13, 14, 15, 16].

ECMO is resource‐intensive with significant associated risks and thus is only offered at specialized centers. To maximize benefits while minimizing harm, careful selection is crucial when deciding which patients should be offered ECPR. To guide this decision, inclusion criteria have emerged, such as time from arrest to hospital‐arrival of less than 30 min and time from arrest to cannulation of less than 60 min, although variation in eligibility criteria exists [6, 17, 18]. It is important to note that the maximum acceptable time from arrest to arrival and arrest to cannulation remains unknown and is highly dependent on patient selection, system structure, and team availability. Despite widespread adoption, there is evidence that a 30‐min arrest to arrival benchmark is difficult to achieve and excludes a significant proportion of the population [17, 18, 19, 20].

Prior limited investigations have modeled ECPR access in the U.S. on a broad national scale or relied upon historic OHCA data to represent event occurrence. The enormous heterogeneity of geography, transportation infrastructure, and ECPR systems across the United States, as well as the relatively low frequency of OHCA, represents a significant barrier to informed development and implementation of local policies [18, 21, 22]. ECPR delivery models must be customized to the specifics of their geographic area and require a granular, comprehensive understanding of region‐specific landscapes and systems.

Therefore, we sought to model current time‐dependent access to ECPR in the City of Boston using geospatial analysis and ground transportation matrices incorporating the dynamic impact of time on scene and local traffic patterns.

## 2 Methods

The data that support the study findings are available from the corresponding author upon reasonable request.

### 2.1 Study Design and Setting

We conducted a geospatial and drive‐time analysis study within the city limits of Boston, Massachusetts to estimate current time‐dependent access to ECPR for OHCA. In Boston, a five‐hospital consortium of ECMO‐capable centers has collaborated with the City of Boston Emergency Medical Systems (Boston EMS) on shared definitions and protocols regarding transportation to an ECPR‐capable center and ECPR eligibility for patients with OHCA [23]. Among the inclusion and exclusion criteria for ECPR in the City of Boston (Table 1) is a time from arrest to arrival at an ECPR‐capable hospital of less than 30 min.

**TABLE 1:** City of Boston ECPR Criteria.

Inclusion criteria	Exclusion criteria
Age 18–70	DNR/DNI status
Witnessed arrest	Uncontrollable hemorrhage
CPR initiated within 10 min of arrest (including bystander CPR) Initial rhythm was shockable (VF or pulseless VT)	Noncardiac cause of arrest Penetrating trauma Burn Hanging Overdose
No ROSC after 3 defibrillation attempts (including AED) Body habitus conducive to ECMO cannulation (LUCAS capable OR smaller) Arrival at receiving facility within 30 min of arrest	Major pre‐existing comorbidities Significant lung disease on home oxygen End‐stage renal disease on dialysis Liver disease with clinical evidence of cirrhosis Pre‐existing major neurological disability Active malignancy Severe pulmonary hypertension on parenteral therapy ○ (epoprostenol, treprostinil) Terminal heart failure and not a candidate for advanced therapies

Calculations used publicly available emergency department location (GPS) coordinates, 2023 American Community Survey 5 Year Data [24, 25], and a readily available traffic network software and application programming interface [ArcGIS; Esri, Redlands, CA], as well as local and national EMS metrics. ArcGIS is a software application and programming interface with comprehensive historical and live street traffic data.

### 2.2 Data Acquisition and Variables

We utilized representative proxy sites for OHCA location corresponding to population‐weighted centroid coordinates within each 2020 U.S. Census Block Group in the City of Boston [26, 27]. A block group is a division of the census tract, generally containing between 600 and 3000 people [28]. This proxy site approach reduced limitations inherent in using historic OHCA data, as OHCA is a relatively low frequency occurrence, and prior events may inadequately predict future event distribution. Locations for the five Boston ECPR centers were defined as the GPS coordinates for the emergency department (ED) ambulance entrance using Google Maps. All coordinates used the NAD83 Massachusetts Mainland geographic coordinate reference system.

To represent time from 9‐1‐1 call to EMS transporting unit arrival, we imputed a fixed EMS dispatch‐to‐scene arrival time of 7.4 min based on the historical Boston EMS median time for priority responses [29]. Within Boston EMS, a priority response designates a time sensitive, life threatening, or potentially life‐threatening emergency. Ambulances are often dispatched from sites other than fixed bases, particularly during times of limited system capacity, limiting the utility of mapping actual distances between EMS stations and OHCA proxy sites.

For time spent on‐scene, we planned a priori to use the 50th percentile, 25th percentile, and 10th percentile for EMS scene time for cardiac arrest calls derived from the 2023 National Emergency Medical Services Information System (NEMSIS) Public‐Release Research dataset. We examined scene times for ground EMS units responding to a 9‐1‐1 call for a cardiac arrest in urban New England that resulted in transport to the ED; this metric is inclusive of time used to move patient from the location of their arrest to the ambulance. In the 6061 encounters meeting these criteria, these times came to 23.9 min, 17.5 min, and 13.0 min, respectively [30].

### 2.3 Drive Time Analysis

We modeled the ground transportation time from the OHCA proxy sites to each ECPR‐capable hospital and selected the minimum drive time for each proxy site. We performed this process twice, varying traffic congestion using ArcGIS. Traffic patterns were modeled using binary levels of “high” versus “low” congestion level to illustrate the range of impact. “High” traffic congestion level was set in ArcGIS as 8 am on a weekday, while “low” traffic congestion level was set as 3 am on a weekday, based on review of historical traffic peak and nadir data for greater Boston [31]. We performed a sensitivity analysis calculating drive times using Mapbox Matrix API [Mapbox, San Francisco, CA].

We then determined geography‐dependent access to ECPR for each OHCA proxy site based on the summative time using the formula [Dispatch‐to‐Arrival Time] + [On‐Scene Time] + [Transport Time to ECPR Center] to evaluate whether the total time fell within the 30‐min arrest to arrival criterion. If this criterion was met for an OHCA proxy site, that corresponding block group was considered to have ECPR access. Using R (Software R version 4.3.1) [32], we visualized results characterizing block groups with and without time‐dependent eligibility for ECPR according to varying scene‐time and traffic congestion conditions with choropleth maps.

We also translated ECPR access from geographic terms to population terms by summing the number of people in each eligible block group, in total and aged 18–69 years, and reporting this as a proportion of Boston's population, in total and aged 18–69. We used this age range as it is the census data point most closely corresponding to current Boston city‐wide ECPR age‐based eligibility criteria (18–70) and more precise age disaggregated data are not included in the census results.

Finally, we calculated the arrest to arrival time as the dependent value in the hypothetical circumstance where 90% of Boston's block groups had time‐dependent access to ECPR. We calculated this under two conditions: “optimal” and “sub‐optimal”, which we defined as [Lowest Traffic +10th Percentile On‐Scene Time] and [Highest Traffic +50th Percentile On‐Scene Time], respectively.

This investigation was reviewed by the Mass General Brigham Institutional Review Board and was determined to be exempt from review as it represented nonhuman subject research.

## 3 Results

**FIGURE 1:**
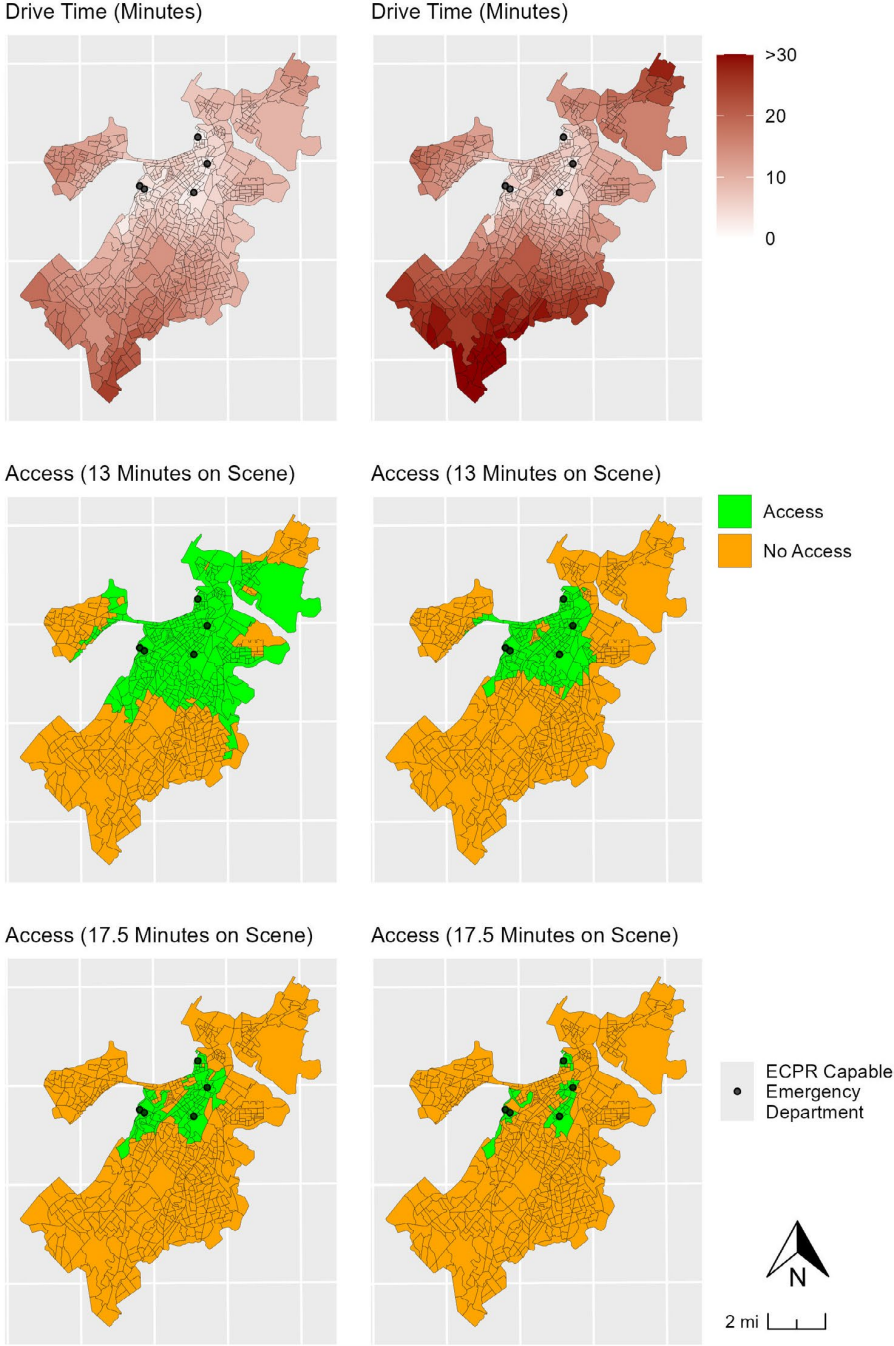
Arrangement of block groups within the City of Boston.

In the City of Boston, there are 581 block groups according to the most recent 2020 Census data [28]. We excluded the two block groups representing the Boston Harbor Islands and the Charles River as the former has no vehicular access and the latter has zero population, resulting in an analytic region of 579 block groups. Figure 1 shows the distribution of block groups within the City of Boston.

**FIGURE 2:**
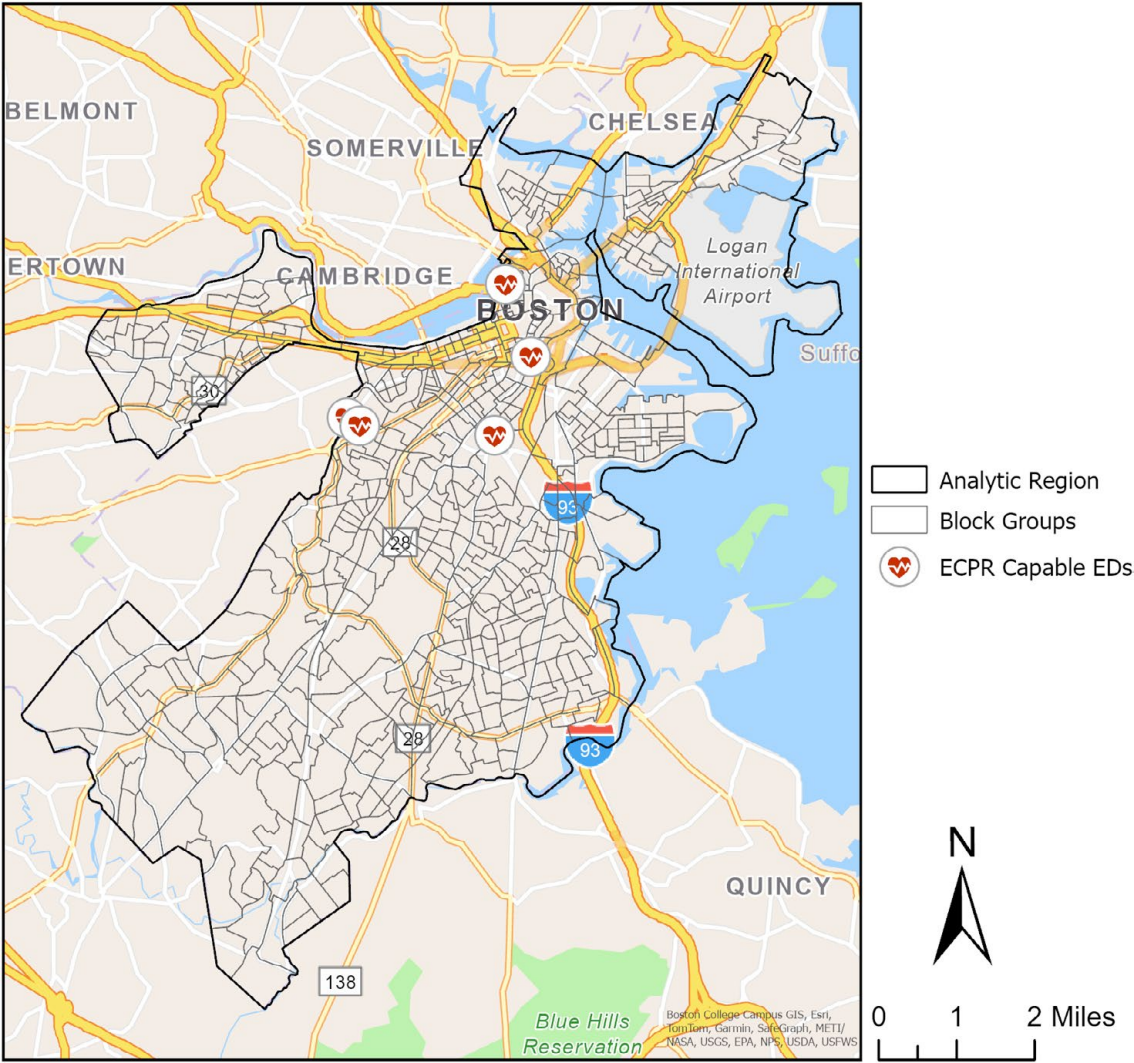
Graphic showing ECPR access based on time spent on scene and level of traffic. *Maps showing median time spent on scene (23.9 min) for either traffic condition are not shown as no block groups had access under those conditions.

The mean population of each block group was 1146 people (SD = 602), and the median was 1049 (IQR = 670). Including just those individuals aged 18–69, the mean population was 847 (SD = 491), and the median was 795 (IQR = 526). Using the 50th percentile for on‐scene time (23.9 min) and the median dispatch‐to‐arrival time (7.4 min) excluded the entire city of Boston from ECPR access under the current time‐dependent eligibility criterion, regardless of traffic patterns. Using the 25th percentile for on‐scene time (17.5 min) and lowest traffic congestion level, 90 census blocks (16%), or 18% of Boston's population aged 18–69 years, could achieve the 30‐min arrest to arrival time. Using the highest traffic congestion levels, access dropped to 36 census blocks (6%), or 7% of Boston's population aged 18–69 years. Using the 10th percentile for on‐scene time (13.0 min) and lowest traffic congestion level, 318 census blocks (55%), or 57% of Boston's population aged 18–69 years, could achieve the 30‐min arrest to arrival time. Using the highest traffic congestion levels, access dropped to 163 census blocks (28%), or 31% of Boston's population aged 18–69 years (Table 2 and **Figure** **2**).

**TABLE 2:** Percentage of population eligible for ECPR by time spent on scene and traffic level.

Time on‐scene	Block groups eligible percent (number)	Total population in eligible block groups percent (number)	Population aged 18–69 in eligible block groups percent (number)
50th Percentile (23.9 min)			
High Traffic	0% (0/579)	0% (0)	0% (0)
Low Traffic	0% (0/579)	0% (0)	0% (0)
25th Percentile (17.5 min)			
High Traffic	6% (36/579)	7% (44,688)	7% (35,434)
Low Traffic	16% (90/579)	17% (110,172)	18% (88,937)
10th Percentile (13.0 min)			
High Traffic	28% (163/579)	29% (190,219)	31% (155,477)
Low Traffic	55% (318/579)	54% (360,258)	57% (288,068)

Sensitivity analysis using Mapbox Matrix API software for the same analyses yielded results highly correlated with those using ArcGIS, suggesting reliability of these methods (Pearson Correlation Coefficient = 0.95).

Achieving time‐dependent ECPR access for 90% of Boston's block groups under previously‐defined “favorable” conditions (lower traffic and 10th percentile on‐scene time) would require 36 min from arrest to arrival at ECPR‐capable center. Achieving this under sub‐optimal conditions (higher traffic and 50th percentile on‐scene time) would require 55.8 min from arrest to arrival at ECPR‐capable center. The distributions of drive times among Boston block groups under both high and low traffic conditions are shown in Supplemental Table S1.

## 4 Discussion

In this geospatial analysis of time‐based ECPR access, we found that the entire population of Boston was excluded under median dispatch‐to‐arrival and EMS on‐scene time conditions with the current city‐wide protocol. Even when modeling favorable time‐on‐scene metrics, only a minority of the city achieved time‐based eligibility for ECPR. These results are surprising in Boston, which is a geographically small, dense city with a large number of ECPR‐capable hospitals per capita. This suggests that access to ECPR in geographically bigger, sprawling cities with fewer ECPR‐capable hospitals may be even more limited. Modeling hypothetical conditions where 90% of Boston block groups are included in ECPR access required arrest to arrival times of almost double the current 30‐min criterion under high traffic conditions. Notably, this 55.8‐min arrest to arrival scenario utilized median on‐scene times for the model, suggesting that a significant population is still excluded from ECPR access with any greater‐than‐average scene time. This reveals an opportunity in the City of Boston for innovation, policy, and protocol reconsiderations if we seek wider implementation of this resource.

In a recent simulated OHCA ECPR case, which was designed to test the Boston EMS‐hospital consortium as part of a quality improvement project, time from arrest to arrival was 29 min despite a dedicated, pre‐staged ambulance crew and a simulated OHCA scene just two blocks from the ECPR‐center [23, 33], suggesting internal validity to our mapped variables, while the reported mean on‐scene time in the ARREST trial was 22.5–23 min, corresponding closely to our 50th percentile time of 23.9 min [34]. One recent observational cohort study of patients with refractory ventricular fibrillation OHCA within the Los Angeles County EMS regional ECPR program found that the median time from 9‐1‐1 call to arrival at an ECPR center was 43 min, lending external validity to the prehospital times seen with our model [35].

Levers for optimizing OHCA care and ECPR access are manifold and may include personalizing the prehospital approach to real‐time geospatial conditions, novel ECPR delivery models, and system‐specific data‐driven eligibility criteria. Predictive modeling, as we present here, offers a practical, cost‐effective initial measure to inform which of these levers is most likely to offer benefit to a particular system. This modeling can clarify which regions fall outside of even the most optimistic ECPR catchment area estimations, more precisely guiding prehospital clinicians in either choosing high‐quality, dedicated, on‐scene resuscitation or prioritizing rapid transport to an ECPR center [21]. Studies not accounting for ECPR adoption showed that continued on‐scene resuscitation was associated with increased survival when compared with intra‐arrest transport, so decreasing on‐scene time in favor of rapid transport would be a departure from standard practice for many EMS systems and would require careful consideration of implications prior to any protocol modification [36, 37].

Although data support policies that use an upper limit of time from collapse‐to‐cannulation of less than 60 min, clinical equipoise remains regarding the 30‐min arrest to arrival time criterion. Some sites use 45‐min for this limit, suggesting an opportunity for customization based on local systems and their efficiency [6, 17, 38]. Although limited by low event occurrence, the LA regional study demonstrating an average time of 43 min from 9‐1‐1 call to ECPR center arrival showed a 27% survival rate among those receiving ECPR. All of these survivors were neurologically intact at discharge despite a median time from cardiac arrest to ECMO of 76 min (IQR 53–94), suggesting further uncertainty in prescribing strict time‐based eligibility criteria [35]. Our methods, coupled with site‐specific ECMO cannulation time metrics, could inform the creation of local arrest to arrival criteria that best optimize ECPR access. Calls to address these time barriers have led to novel ECPR delivery programs and innovative transport approaches, which show promise in predictive models for reducing geography‐based access inequities [18, 22].

Boston primarily utilizes hospital‐based ECPR initiation, requiring that OHCA patients be transported from the field to the designated hospital for ECPR cannulation to occur. Although this is the most common model, this method is associated with smaller geographic catchment areas and high rates of time‐based exclusion, as seen in our investigation [39]. This approach has successfully been implemented in other regions by piloting prehospital initiation or a rendezvous method, both of which shorten transport‐to‐cannulation time by dispatching ECPR teams directly to the field [39, 40, 41]. Prehospital ECPR, as the name suggests, dispatches ECMO teams directly to the OHCA patient and has been successfully implemented in numerous settings [12, 42]. In the rendezvous approach, the OHCA patient is taken to the nearest hospital while an ECPR team is simultaneously deployed to that hospital where the patient is cannulated onto ECMO and subsequently transferred to the ECMO center [39, 40, 41].

In rural or peri‐urban settings, varying modes of EMS transport, including helicopter‐based systems, may further reduce time to cannulation [22]. These approaches effectively increase the geographic spheres of access surrounding ECPR‐capable centers. Applying our methods to the various regions with ECPR capability may offer insight into whether investment in local prehospital or rendezvous ECPR infrastructure should be explored [6, 17, 38].

There are several limitations to the current study. First, modeling is inherently an imperfect prediction of reality. Although we used historic, data‐based statistics and models for dispatch‐to‐arrival time, on‐scene time, and scene‐to‐hospital time, these are approximations and may vary in real practice. Importantly, we did not have access to collapse‐to‐EMS dispatch data. Dispatch to arrival time especially is difficult to predict given that available ambulances are deployed from their current location at the time of a 9‐1‐1 call, which cannot be predicted in advance. Similarly, the historic EMS data we used to inform our models may not reflect current EMS practice. EMS protocols have historically prioritized on‐scene care for OHCA rather than expediting intra‐arrest transport [37]. However, our use of the 10th and 25th percentile scene times was intentional to better reflect practice when emphasizing rapid transport for ECPR. Additionally, ambulances may drive faster than traffic when using lights and sirens, although the time savings in urban areas is relatively small and is not likely to bias the results [43, 44]. Modeling also does not specifically account for variables such as inclement weather or staffing‐based disruptions in ECMO team capability.

Second, our investigation is limited to time‐based criteria for ECPR eligibility, which is a small fraction of the variables, such as age, comorbidities, and BMI that are considered in the decision of whether to offer ECPR to a patient. This limitation, however, was intentional as it focuses on one of the only modifiable factors in eligibility and is amenable to targeted innovation, such as prehospital ECPR, rendezvous ECPR, and varying modes of EMS transport. In this study, we included a population between 18 and 69 years old, as this is the most granular data available within the census data, which may slightly underestimate eligibility by age group but almost certainly overestimates eligibility when accounting for excluded comorbidities.

Lastly, we focused on the specific geographic context of Boston, and our findings cannot comment on ECPR access outside of these city limits. Given the wide variety of geography and traffic networks serving ECPR centers nationally, we felt this focused scope was necessary to meaningfully describe access for this population. Although we do not consider our results generalizable to other cities or to peri‐urban and rural communities, we do believe our methods are generalizable and can be replicated to characterize all regions across the U.S. Additionally, utilizing block groups as a proxy site for cardiac arrest does not model dynamic movement of individuals and thus introduces potential bias in that these population‐weighted regions are weighted based on primary residence location. Although this may be a limitation, utilizing census block groups does account for the entirety of Boston's geographic footprint, except for the two block groups described in our methods that we intentionally excluded due to their lack of occupants.

## 5 Conclusions

ECPR is a promising, specialized, and resource‐intensive technology with evidence supporting improved outcomes after OHCA when implemented by high‐performing teams under specific circumstances. Access to ECPR is limited, in large part by hyper‐local traffic and geospatial forces impinging upon critical time‐dependent criteria for eligibility. In this study, we found that existing protocols for Boston prevent almost the entire city's population from accessing ECPR under typical conditions. This investigation adds to the scant literature describing the current landscape of ECPR access, offers greater insight for clinical practice, and provides potential direction for future exploration. We anticipate that these methods may be replicated and made portable for application in other communities where ECPR is available. Further study should evaluate the in vivo authenticity and external generalizability of these models, explore their potential to inform targeted innovation in ECPR delivery, and determine how to best use modeling to optimize equity and scope of ECPR access.

## Funding

This work was supported by Mass General Brigham.

## Conflicts of Interest

The authors declare no conflicts of interest.

## Supporting information


**Table S1:** Distribution of drive times (in minutes) among the 579 Boston block groups.

## Data Availability

The data that support the findings of this study are available from the corresponding author upon reasonable request.
