# Supplementary material for: Determining Access for a City‐Wide Extracorporeal Cardiopulmonary Resuscitation (ECPR) Initiative Using Geospatial Analysis
Source: Acad Emerg Med. 2026 Apr 13;33(4):e70288. doi: 10.1111/acem.70288 (PMC13075459; doi:10.1111/acem.70288)
Supplement: Supplementary file 1 — Table S1: Distribution of drive times (in minutes) among the 579 Boston block groups. [file ACEM-33-0-s001.docx]

**Supplemental Table 1.** Distribution of drive times (in minutes) among the 579 Boston block groups.

|  | **Traffic Conditions** | |
| --- | --- | --- |
| **Percentile** | Low | High |
| 10% | 4.3 | 5.8 |
| 25% | 6.3 | 9.0 |
| 50% | 9.1 | 13.5 |
| 75% | 12.9 | 20.7 |
| 90% | 15.6 | 25.4 |
